# A longitudinal model for the Mayo Clinical Score and its sub-components in patients with ulcerative colitis

**DOI:** 10.1007/s10928-021-09789-2

**Published:** 2021-10-16

**Authors:** Sonoko Kawakatsu, Rui Zhu, Wenhui Zhang, Meina T. Tang, Tong Lu, Angelica L. Quartino, Matts Kågedal

**Affiliations:** 1 Clinical Pharmacology, Development Sciences, Genentech Inc., 1 DNA Way, South San Francisco, CA USA; 2 Thomas J. Long School of Pharmacy, University of the Pacific, 3601 Pacific Avenue, Stockton, CA USA; 3 Present Address: Clinical Pharmacology and Quantitative Pharmacology, AstraZeneca, Gothenburg, Sweden; 4 Present Address: Metrum Research Group, Tariffville, CT USA

**Keywords:** Ulcerative colitis, Placebo effect, Mayo Clinical Score, Proportional odds, Categorical modeling, NONMEM

## Abstract

Clinical trials in patients with ulcerative colitis (UC) face the challenge of high and variable placebo response rates. The Mayo Clinical Score (MCS) is used widely as the primary endpoint in clinical trials to describe the clinical status of patients with UC. The MCS is comprised of four subscores, each scored 0, 1, 2 and 3: rectal bleeding (RB), stool frequency (SF), physician’s global assessment (PGA), and endoscopy (ENDO) subscore. Excluding the PGA subscore gives the modified MCS. Quantitative insight on the placebo response, and its impact on the components of the MCS over time, can better inform clinical trial design and interpretation. Longitudinal modeling of the MCS, and the modified MCS, can be challenging due to complex clinical trial design, population heterogeneity, and limited assessments for the ENDO subscore. The current study pooled patient-level placebo/standard of care (SoC) arm data from five clinical trials in the TransCelerate database to develop a longitudinal placebo response model that describes the MCS over time in patients with UC. MCS subscores were modeled using proportional odds models, and the removal of patients from the placebo/SoC arm, or “dropout”, was modeled using logistic regression models. The subscore and dropout models were linked to allow for the prediction of the MCS and the modified MCS. Stepwise covariate modeling identified prior exposure to TNF-α antagonists as a statistically significant predictor on the RB + SF subscore. Patients with prior exposure to TNF-α antagonists had higher post-baseline RB + SF subscores than naive patients.

**Supplementary Information:**

The online version contains supplementary material available at 10.1007/s10928-021-09789-2.

## Introduction

Ulcerative colitis (UC) is a type of inflammatory bowel disease characterized by chronic inflammation of the large intestine. The clinical course is unpredictable, and is characterized by periods of remission and relapse. Currently, there is no cure for UC, and treatment is focused on minimizing symptoms and disease progression. Clinical trials for the development of new treatments in this population face the challenge of high and variable placebo response rates, which makes it difficult to draw conclusions about treatment-related response. In addition, evaluating efficacy of a new agent in pediatric trials encounters severe ethical and feasibility issues if including a placebo/standard of care (SoC) arm. When there is already efficacy data for an active treatment in adults, the use of a placebo/SoC arm in pediatric trials is avoided because it would expose patients to a known inferior treatment. Enrollment in a pediatric trial would also be difficult due to the lower prevalence of UC in children [1–4]. Developing a model to describe longitudinal placebo response can help guide the design of clinical trials (e.g. sample size calculations), and could potentially provide information about expected placebo response in trials where a placebo/SoC arm is not available. In addition, such a model would allow for the evaluation of intrinsic and extrinsic factors that can influence placebo response in patients with UC.

TransCelerate BioPharma is an organization formed for biopharmaceutical member companies to collaborate and address inefficiencies in drug development. To promote the understanding of placebo response and natural histories of disease, TransCelerate Biopharma launched the Historical Trial Data Sharing (Controls) initiative to share and pool placebo/SoC arm data from completed clinical trials [5]. The database is readily accessible by member companies and contains de-identified, patient-level placebo/SoC arm clinical trial data for various indications including UC. The current study extracted data from this database to develop a placebo response model.

The Mayo Clinical Score (MCS) has been widely used in clinical trials to describe the clinical status of patients with UC. The MCS consists of four subscores, each scored 0, 1, 2 and 3: rectal bleeding (RB), stool frequency (SF), physician’s global assessment (PGA), and endoscopy (ENDO) subscore [6]. The subscores are added to give the MCS, which can range from 0 to 12. Higher scores indicate increased disease severity. Excluding the ENDO subscore from the MCS gives the partial MCS, and excluding the PGA subscore gives the modified MCS [7, 8]. The utility of the PGA subscore has been called into question, as it is unclear what distinct information it provides over the other components of the MCS. This subscore is also subjective and can introduce variability in the data [7]. Therefore, the use of the MCS as an endpoint for pivotal trials is no longer recommended [9, 10]. Instead, regulators recommend the use of the modified MCS. The development of a longitudinal model for the modified MCS can be challenging due to the limited availability of the repeated ENDO subscores given the ENDO subscore requires an invasive procedure. Typically a colonoscopy is performed at the time of randomization, time of the primary endpoint, and selected times during the maintenance phase. While timepoints for ENDO subscore are limited, the other non-invasive subscores are evaluated more frequently and can provide longitudinal data to support estimation of the time course of placebo response.

Modeling the time course of the modified MCS faces the additional challenge of complex trial design. For studies with both an induction and maintenance phase, patients in the placebo/SoC arm may be removed from the trial at the end of induction or may progress to the maintenance phase through a variety of mechanisms. Patients that progress into the maintenance phase may remain in the placebo/SoC arm, be re-randomized into a placebo/SoC or treatment arm, or be placed into a treatment arm based on responder status. A model accounting for this potential removal of patients from the placebo/SoC arm at the end of induction would allow for a more accurate characterization of longitudinal placebo response through the maintenance phase. Because an investigational therapy must demonstrate efficacy in both the induction and maintenance phase, a better understanding of placebo response in the maintenance phase is needed to aid the evaluation of drug effect.

Endpoints from clinical trials in UC are often categorical in nature, and these categorical variables can be nominal or ordinal. The modified MCS is ordered categorical due to the finite number of integer values that have a clear order in the scale. Ordered categorical data that have greater than or equal to 10 categories are typically modeled as continuous data due to the large number of parameters associated with the categorical approach. With a large enough dataset, however, the ordered categorical approach may be appropriate for modeling the modified MCS. It may be preferred so that predictions will always reflect actual scores, whereas a continuous model may predict partial scores that are inconsistent with the actual modified MCS that can be obtained. Recently, the MCS for golimumab in patients with UC was modeled using continuous and ordered categorical approaches [11]. In that analysis, the model developed using the ordered categorical approach produced more accurate predictions than the continuous model. The proportional odds (PO) model structure was first used for nonlinear mixed effects modeling by Sheiner, and has since been used widely to characterize ordered categorical data [12]. Therefore, the current study used a PO model to describe the longitudinal modified MCS. The modeling of the PGA subscore, to allow for predictions of the MCS, was also explored.

The previously developed categorical model describes the MCS as a single endpoint [10]. The aim of the current analysis is to develop a longitudinal model that describes the subscores of the MCS as separate endpoints during both induction and maintenance phases of a clinical trial in UC. This would provide the flexibility to model individual subscores and various combinations of the subscores (e.g. MCS, modified MCS, partial MCS). The current analysis also aims to improve model estimates in the maintenance phase by accounting for the complex clinical trial design in UC, where patients may be removed from the placebo arm at the end of the induction phase. The effect of intrinsic and extrinsic factors on the MCS subscores were also evaluated. The resulting model would provide detailed information on the relationship between the subscores, and the longitudinal behavior of the MCS, modified MCS, or partial MCS during both induction and maintenance phases of a clinical trial.

## Methods

### Placebo/SoC arm database

All available longitudinal, patient-level placebo/SoC arm data from clinical trials for UC were extracted from the TransCelerate database. Data were pooled from five randomized, double-blind, placebo-controlled, multicenter phase 2 and 3 trials for moderate to severe active UC [13–17]. The modeling dataset was assembled and visualized using the statistical software R (version 3.6.0).

### Model structure

A longitudinal model for the RB + SF subscore was developed initially, as the RB + SF subscore data is richer than the ENDO subscore data. This allowed for subsequently developed subscore (i.e. ENDO and PGA subscores) and dropout models to be informed by the RB + SF subscore model. The linkages between these separate model components were explored to develop a complete model that can estimate the modified MCS or MCS over time. The predicted modified MCS or MCS would be derived as the sum of the separate predictions of the appropriate subscore models.

#### Subscore models

The subscores of the MCS were modeled using several PO models. Each PO model estimates the cumulative probability of having an observation *Y*_i,j_ for the ith patient at the jth timepoint, that is greater than or equal to a given score *m*. Logit transformations were used to ensure probabilities fell within the range from 0 to 1. The general PO model structure can be represented by the following equation:

1 $$Logit[P({Y}_{i,j}\ge m|{\eta }_{i}]= {\alpha }_{m}+ PLB + {\eta }_{i}$$

α_m_ is the intercept, PLB is the placebo effect, and η_i_ is the interindividual variability (IIV). In the model, P(Y_i,j_ ≥ 0) = 1, and α_m_ for m > 1 was reparameterized using a value DF_m_ < 0 to ensure that P(Y_i,j_ ≥ m) > P(Y_i,j_ ≥ m + 1). DF_m_ is the difference between α_m_ and α_m-1_.

2 $${\alpha }_{m}= {\alpha }_{m-1}+ {DF}_{m} \, [\mathrm{for\ m}>0]$$

Baseline values for the subscores were included as covariates on the intercept parameter of the respective subscore model. Additionally potential effects of baseline covariates: prior exposure to TNF-α antagonists, age, c-reactive protein (CRP), albumin, smoking status, concomitant medications, and steroid use were evaluated on the intercept parameter using a stepwise covariate modeling (SCM) approach. Covariates that resulted in a reduction in the objective function value (OFV) of greater than 3.84 (p < 0.05 for one additional degree of freedom) were retained in the forward selection, and those that resulted in a change in OFV of greater than 10.8 (p < 0.001) were retained in the backward elimination. Because individual-level information on concomitant medications and steroid use were not readily available for model building, summary-level information for each study on concomitant medications required before baseline and permitted during the study, and the proportion of placebo/SoC arm patients using steroids at baseline were used for covariate modeling. Studies were assigned a categorical value based on study design criteria related to concomitant medications, and this value was directly assigned to patients within the study. Similarly, the proportion of patients using steroids at baseline was identified in each study, and this value was directly assigned to patients in that study as a continuous covariate (e.g. if a study had 50% of patients using steroids at baseline, patients in that study were assigned a value of 0.5) for covariate modeling.

#### Dropout model

Dropout was modelled to enable simulations that can be compared to the observed data. For the trials that included a maintenance phase, a dropout model was implemented to estimate the loss of patients between the induction and the maintenance phase. In addition, the gradual removal of patients over time during the maintenance phase was estimated separately in the model. A logistic regression model structure was used for the dropout model.

### Model building and evaluation

Model building was done using a non-linear mixed effects approach in the NONMEM software version 7.4.3. The Laplacian estimation method with the likelihood option in the estimation record was used for parameter estimation. Model selection was based on changes in OFV. Model performance was evaluated using visual predictive checks (VPCs) in which 500 replicates of the dataset were simulated and compared to observed data. The VPC simulations were based on complete observation time-points in all patients (i.e. up to the end of study). Observations after the simulated time of drop-out were censored to achieve data that could be compared to the observed results. The simulated and observed scores shown in the VPCs are based on the patients remaining in the study (i.e. that did not drop out). Imputation at planned visits was done to provide records that allow for the model to predict the probability of dropout at any planned visit, rather than limiting model predictions to observed visits for a given individual. Without this imputation, a patient during simulation would automatically dropout at the observed time of dropout or earlier. The imputed rows contained negative values that indicate a patient has dropped out, and these values are not included in calculating the observed or predicted average scores. Bootstrapping was performed to evaluate parameter uncertainty. Model diagnostics and SCM were assisted by R version 3.6.0, Perl-speaks-NONMEM (PsN) toolkit version 4.9.0 and Pirana version 2.9.9

## Results

### Placebo/SoC arm database

Individual-level longitudinal data from 755 adult patients, placebo/SoC arm, were pooled from five Phase 2/3 clinical trials [13–17]. The trials were conducted during the years 2006 to 2011. Three of the studies were both induction and maintenance phase studies, and two of the studies were induction phase only. All placebo/SoC arm patients with data in the maintenance phase were also in the placebo/SoC arm during induction phase. Patients had moderate to severe active UC, as evidenced by a MCS of 6 to 12 points and ENDO subscore of 2 to 3 points at baseline. Across the trials, the RB, SF, and PGA subscores were evaluated every 2–6 weeks, and ENDO subscore was evaluated at weeks 0, 8, 32/36, and 52. Summary of clinical trial and patient characteristics are summarized in Table 1.

**Table 1:** Baseline characteristic of studies and patients included in modeling analysis

Sponsor and ClinicalTrials.gov number	Abbvie	Abbvie	Abbvie	BMS	Pfizer	Total
	NCT00385736	NCT00408629	NCT00853099	NCT00410410	NCT00787202	
Study characteristics						
N (induction/maintenance)	222/–^a^	256/143	96/57	135/20	46/–^a^	755/220
Induction phase duration (weeks)	8	8	8	12	8	
Maintenance phase duration (weeks)	–	44	44	40	–	
Patient baseline characteristics						
Age, years, mean ± SD	39.7 ± 12.66	41.4 ± 13.13	41.3 ± 13.56	41.2 ± 13.23	42.0 ± 13.93	40.9 ± 13.11
Mayo Clinical Score, mean ± SD	8.8 ± 1.60	8.9 ± 1.73	8.5 ± 1.56	8.7 ± 1.56	8.3 ± 1.46	8.8 ± 1.63
Rectal Bleeding + Stool Frequency Subscore, N (%)	0: 0 (0%) 1: 2 (0.9%) 2: 21 (9.5%) 3: 47 (21.2%) 4: 67 (30.2%) 5: 59 (26.6%) 6: 26 (11.7%) Missing: 0 (0%)	0: 1 (0.4%) 1: 4 (1.6%) 2: 22 (8.6%) 3: 41 (16.0%) 4: 65 (25.4%) 5: 83 (32.4%) 6: 40 (15.6%) Missing: 0 (0%)	0: 0 (0%) 1: 2 (2.1%) 2: 11 (11.5%) 3: 16 (16.7%) 4: 33 (34.4%) 5: 29 (30.2%) 6: 5 (5.2%) Missing: 0 (0%)	0: 0 (0%) 1: 3 (2.2%) 2: 16 (11.9%) 3: 31 (23.0%) 4: 36 (26.7%) 5: 37 (27.4%) 6: 11 (8.1%) Missing: 1 (0.7%)	0: 1 (2.2%) 1: 0 (0%) 2: 6 (13.0%) 3: 14 (30.4%) 4: 12 (26.1%) 5: 11 (23.9%) 6: 2 (4.3%) Missing: 0 (0%)	0: 2 (0.3%) 1: 11 (1.5%) 2: 76 (10.1%) 3: 149 (19.7%) 4: 213 (28.2%) 5: 219 (29.0%) 6: 84 (11.1%) Missing: 1 (0.1%)
Endoscopy Subscore, N (%)	0: 0 (0%) 1: 1 (4.5%) 2: 112 (50.5%) 3: 109 (49.1%) Missing: 0 (0%)	0: 0 (0%) 1: 0 (0%) 2: 138 (53.9%) 3: 118 (46.1%) Missing: 0 (0%)	0: 0 (0%) 1: 0 (0%) 2: 55 (57.3%) 3: 41 (42.7%) Missing: 0 (0%)	0: 0 (0%) 1: 0 (0%) 2: 55 (40.7%) 3: 79 (58.5%) Missing: 1 (0.7%)	0: 0 (0%) 1: 0 (0%) 2: 24 (52.2%) 3: 22 (47.8%) Missing: 0 (0%)	0: 0 (0%) 1: 1 (0.1%) 2: 384 (50.9%) 3: 369 (48.9%) Missing: 1 (0.1%)
PGA subscore, N (%)	0: 0 (0%) 1: 8 (3.6%) 2: 155 (69.8%) 3: 59 (26.6%) Missing: 0 (0%)	0: 1 (0.4%) 1: 16 (6.3%) 2: 161 (62.9%) 3: 78 (30.5%) Missing: 0 (0%)	0: 0 (0%) 1: 4 (4.2%) 2: 73 (76.0%) 3: 19 (19.8%) Missing: 0 (0%)	0: 0 (0%) 1: 5 (3.7%) 2: 94 (69.6%) 3: 36 (26.7%) Missing: 0 (0%)	0: 0 (0 %) 1: 1 (2.2%) 2: 36 (78.3%) 3: 9 (19.6%) Missing: 0 (0%)	0: 1 (0.1%) 1: 34 (4.5%) 2: 519 (68.7%) 3: 201 (26.6%) Missing: 0 (0%)
Prior anti-TNF therapy, N (%)	0 (0%)	102 (40%)	0 (0%)	27 (20%)	12 (26%)	141 (19%)
Baseline steroid use^b^, (%)	67.60%	56.90%	60.40%	44.30%	27%	
Concomitant medications/standard of care	• Aminosalicylates • Azathioprine/ 6-mercaptopurine • Oral steroid	• Aminosalicylates • Azathioprine/ 6-mercaptopurine • Oral steroid	• Aminosalicylates • Azathioprine/ 6-mercaptopurine • Oral steroid	• Aminosalicylates • Azathioprine/ 6-mercaptopurine • Oral steroid	• Aminosalicylates • Oral steroid	

*N* number of patients, *SD* standard deviation

^a^NCT00385736 and NCT00787202 only included an induction phase

^b^Value obtained from summary provided in publications for each clinical trial [13–17]

### Mayo Clinical Score model

**Fig. 1:**
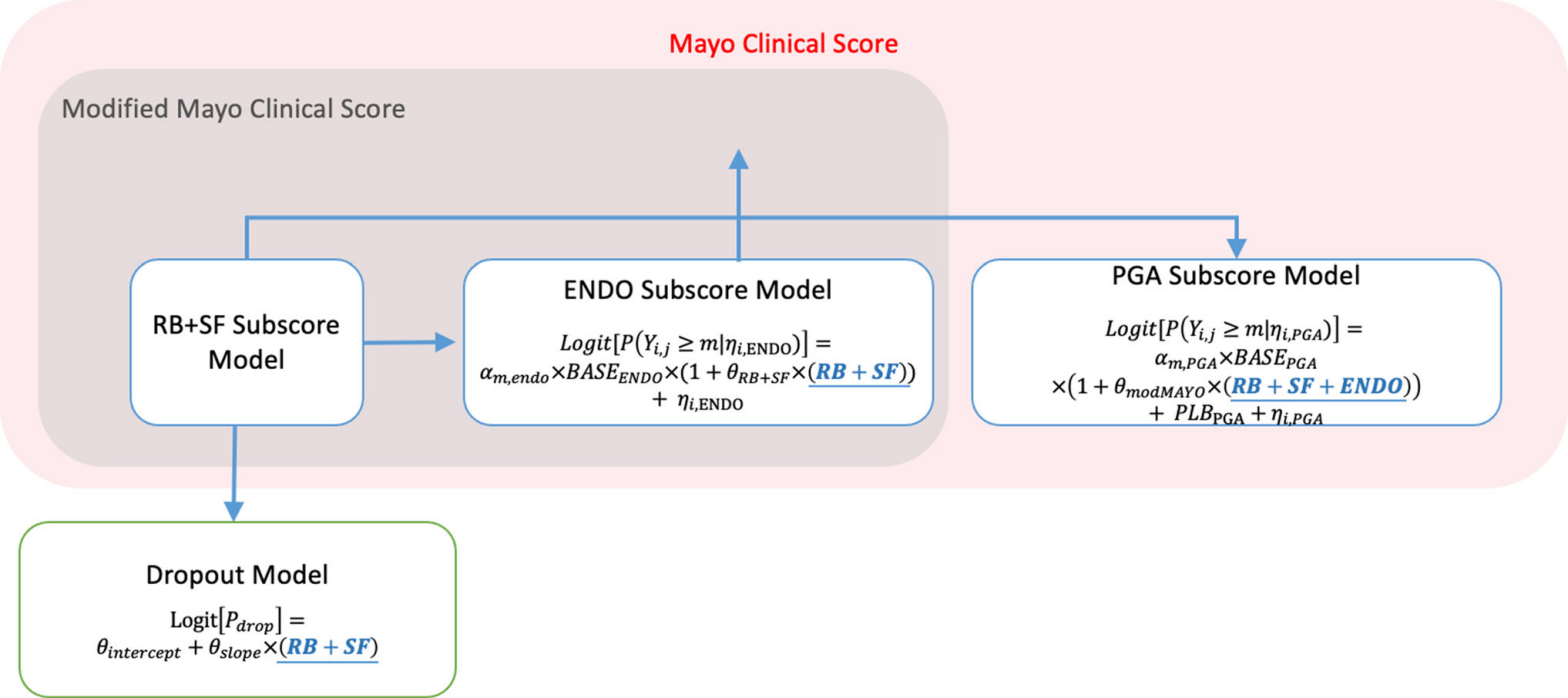
Schematic representing the overall model structure. The RB + SF subscore informs predictions for the ENDO and PGA subscore models, and the dropout model. The ENDO subscore is combined with the RB + SF subscore to give the modified MCS, which informs the PGA subscore model. Equations showing the linkages between models are included in the model scheme

**Fig. 2:**
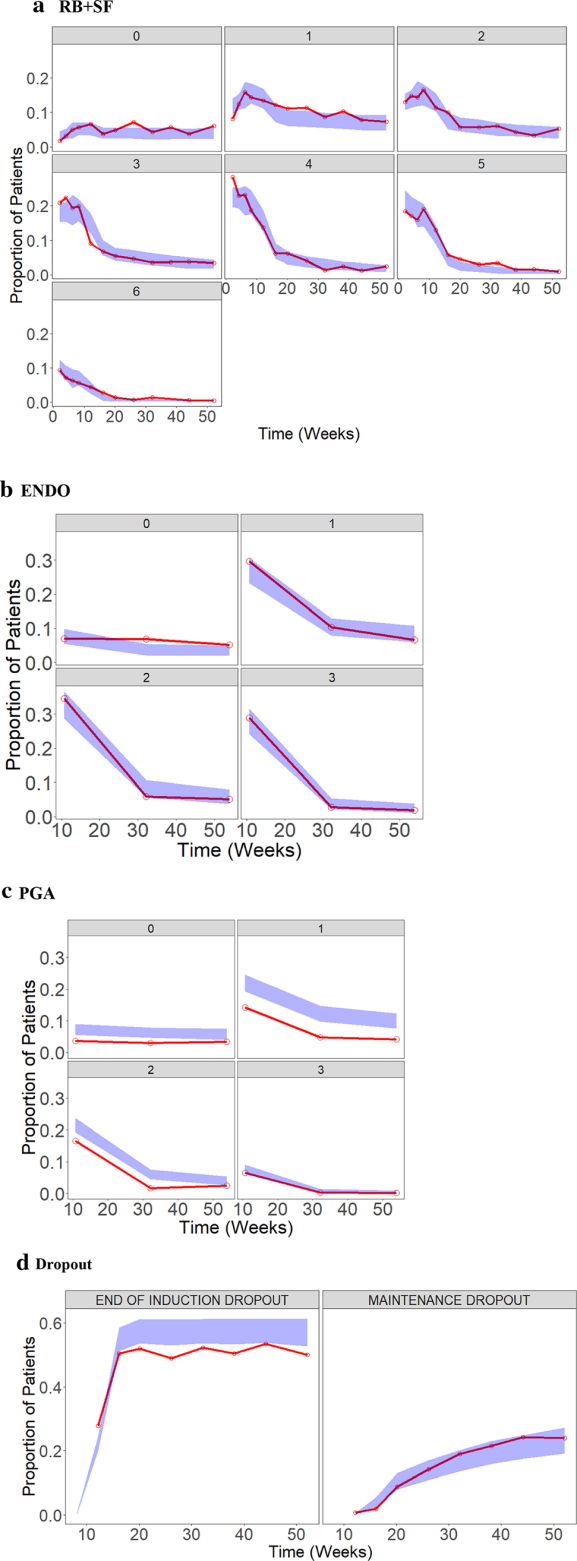
Categorical VPCs are shown for the post-baseline **a** RB + SF subscore, **b** ENDO subscore, **c** PGA subscore, and **d** end of induction and maintenance phase dropout. The observed data are represented by the red line, and are overlaid on top of shaded areas representing the 95% prediction interval by the model. The y-axis is the proportion of remaining patients in each category to the total patients enrolled in the induction/maintenance phase

The complete model consists of three subscore models and one dropout model that are linked together using the RB + SF subscore as a covariate in the ENDO subscore and dropout model, and the modified MCS as a covariate in the PGA subscore model (Fig. 1). The MCS subscores were modeled using a PO model. The RB and SF subscores were summed and modeled as one endpoint (RB + SF). A linear placebo effect was included in the RB + SF subscore model. A linear model was selected as it provided a good description of the data. Estimating separate slopes for induction and maintenance phases as a piecewise linear function resulted in a significant change in OFV (ΔOFV = -81.1), and this was incorporated in the final model (Eq. 6). An exploration of the dataset using the Spearman rank correlation test revealed the RB + SF subscore and ENDO subscore (ρ = 0.65), and the modified MCS and PGA subscore were well correlated (ρ = 0.80). The observed RB + SF subscore from the same or most recent study visit was therefore included as a time-varying covariate in the ENDO subscore model. This resulted in a significant change in OFV (ΔOFV = − 309.4). The ENDO subscore was modeled as a function of the baseline ENDO score and the observed RB + SF subscore. Any time-varying placebo response was hence included indirectly based on the time-varying RB + SF subscore. Attempts to estimate parameters for an independent placebo effect on the ENDO subscore resulted in difficulty with model convergence and therefore could not be estimated. The VPC for the ENDO subscore (Fig. 2b), however shows the model predictions fitting the observed data, suggesting that an additional slope parameter is not needed. The development of a subscore model was also explored for the PGA subscore. The observed modified MCS, derived by adding predictions from the RB + SF and ENDO subscore models, was included as a time-varying covariate in the PGA subscore model. Inclusion of the modified MCS as a time-varying covariate resulted in a significant change in the OFV (ΔOFV = -609.7). The PGA subscore model included a linear placebo effect, similar to the placebo effect included in the RB + SF subscore model. Without this additional placebo effect parameter, the model significantly overpredicted the PGA subscore over time. IIV was estimated for the intercept parameter (α_m_) of each of the subscore models. The dataset did not support the estimation of IIV on the placebo slope parameter. Baseline values of the subscores were included as covariates in the corresponding subscore model. In the SCM, the effect of prior exposure to TNF-α antagonists on the RB + SF subscore model was the only covariate retained. None of the tested covariates were identified as significant on the ENDO subscore model. Patients with prior exposure to TNF-α antagonists had higher post-baseline RB + SF subscores than naïve patients. The subscore models are represented by the following equations:

3 $$Logit[P(Y_{{{\text{i}},{\text{j}}}} \ge m|\eta_{i,RBSF} )] = \alpha_{m,RBSF} \times BASE_{RB + SF} \times TNF + PLB_{RB + SF} + \eta_{i,RBSF}$$

4 $$Logit[P(Y_{i,j} \ge m|\eta_{i,ENDO} )] = \alpha_{m,ENDO} \times BASE_{ENDO} \times \, PDV_{RB + SF} + \eta_{i,ENDO}$$

5 $$Logit[P(Y_{i,j} \ge m|\eta_{i,PGA} )] = \alpha_{m,PGA} \times BASE_{PGA} \times PDV_{\bmod MAYO} + PLB_{PGA} + \eta_{i,PGA}$$

where the baseline RB + SF or PGA subscore (*BASE*) and the RB + SF or modified MCS from the same or most recent visit (*PDV*) were included as continuous covariates with the general structure *(1* + *θ* × *(covariate))*. The effect of prior exposure to TNF-α antagonists (*TNF*) and baseline ENDO subscore (*BASE*_*ENDO*_) were included as categorical covariates with the general structure (*1* + *θ*).

The piecewise linear function for the placebo effect can be written as:

6 $$PLB = - \theta_{induction} \times TIME_{induction} - \theta_{{ma{\text{int}} enance}} \times TIME_{{ma{\text{int}} enance}}$$

The dropout of patients was estimated at the end of the induction phase, as well as during the maintenance phase. Logistic regression with an intercept parameter, and a slope parameter for the effect of the most recent RB + SF subscore on the probability of dropping out was used, with separate parameter estimates for dropout at the end of induction and during maintenance. With this dropout model, dropout does not occur prior to the end of induction. A positive slope was estimated for both the induction and maintenance phase showing that patients with higher RB + SF subscores had an increased probability of dropping out at the end of induction and during the maintenance phase. The dropout model is represented by the following equation:

7 $$Logit[P_{drop} ] = \theta_{{{\text{int}} ercept}} + \theta_{slope} \times (RB + SF)$$

Parameter estimates for the subscore models and dropout model are presented in Table 2. For parameters that can take a positive or negative value, standard error (SE) is presented instead of relative standard error (RSE). Both the SE and RSE in the table were obtained from the NONMEM covariance step. Non-parametric bootstrapping was performed and for most parameters, resulted in SEs of similar magnitude as those provided by the NONMEM covariance step. SE from bootstrapping were slightly larger for the modified MCS covariate parameter and intercept parameters in the PGA subscore model.

**Table 2:** Model parameter estimates

Parameter	Estimate	RSE (%)	SE
RB + SF subscore PO model			
α_1_	5.34		0.19
DF_2_	− 2.34	5.3	
DF_3_	− 1.32	5.3	
DF_4_	− 1.37	5.1	
DF_5_	− 1.66	5.2	
DF_6_	− 2.27	5.9	
SLOPE_PLB,IND_ (1/day)	0.015		0.0020
SLOPE_PLB,MAINT_ (1/day)	0.0011		6.4 $\times$ 10^–4^
BL_RBSF_α1_	0.18		0.013
TNF_α1_	0.14		0.037
Var(η_α1_)	3.47	9.4	
ENDO subscore PO model			
α_1,endo_	0.53		0.17
DF_2,endo_	− 2.84	7.3	
DF_3,endo_	− 2.56	8.0	
BL_ENDO _α1,endo_	0.41		0.073
PDV_RBSF _α1,endo_	1.63		0.59
Var(η_α1,endo_)	1.01	34.1	
PGA subscore PO model			
α_1,PGA_	0.0093		4.4 $\times$ 10^–4^
DF_2,PGA_	− 4.56	9.0	
DF_3,PGA_	− 4.77	10.0	
SLOPE_PLB,PGA,IND_	0.016		0.0039
SLOPE_PLB,PGA,MAINT_	0.0023		8.6 $\times$ 10^–4^
BL_PGA _α1,PGA_	0.24		0.035
PDV_MMCS _α1,PGA_	130		13
Var(η_α1,PGA_)	1.21	57.2	
End of induction dropout logistic regression model			
INTERCEPT_IND_	− 1.94		0.25
SLOPE_IND_	0.68		0.071
INTERCEPT_MAINT_	− 4.65		0.31
SLOPE_MAINT_	0.84		0.072

*RSE* relative standard error, *SE* standard error, *α*_*1*_ intercept parameter on the logit scale for score ≥ 1, *DF*_*k*_ parameter for score k such that α_k_ = α_k-1_ + df_k_, SLOPE_*PLB,IND*_ slope of the time effect on the subscore during induction phase, SLOPE_*PLB,MAINT*_ slope of the time effect on the subscore during maintenance phase, *BL_RBSF* effect of baseline RB + SF subscore, *TNF* effect of prior anti-TNF treatment, *Var(η)* variance of between-subject variability, BL_ENDO effect of baseline ENDO subscore, PDV_RBSF effect of observed RB + SF subscore, BL_PGA effect of baseline PGA subscore, PDV_MMCS effect of observed modified MCS, INTERCEPT_IND_ intercept of the logistic regression at the end of induction phase, SLOPE_IND_ slope of the logistic regression at the end of induction phase, INTERCEPT_MAINT_ intercept of the logistic regression during maintenance phase, SLOPE_MAINT_ slope of the logistic regression during maintenance phase

### Model evaluation

VPCs were conducted such that the ENDO and PGA subscore models generated predictions based on the model-predicted RB + SF and modified MCS, respectively. Categorical VPCs were generated for each of the subscore and dropout models. The VPCs show the proportion of patients in each category over time. The proportions for each of the subscore categories account for dropout patients and are calculated using the number of patients in each category over the total number of patients possible in the induction/maintenance phase. Because the progression of patients into the maintenance phase in induction-only trials is not possible, the denominator is different for the induction and maintenance phase. Categorical VPCs of the RB + SF and ENDO subscore models show an overall good agreement between the predicted 95% confidence interval and observed proportions (Fig. 2). For the PGA subscore, a PO model informed by the modified MCS slightly overpredicted the proportion of patients with subscores of 1 and 2 (Fig. 2). Timepoints start at week 10 for ENDO and PGA due to the first post-baseline ENDO assessment occurring during week 8–12 in the trials. While the dropout model appears to fit the observed data well during the maintenance phase, the model appears to overpredict the proportion of patients dropping out at the end of induction (Fig. 2). The dropout model estimates dropout at a single time point at the end of induction, and the appearance of fluctuations during maintenance phase in the VPC is a result of data binning and differences between studies in scheduled visits. It should also be noted that the proportions for the ENDO and dropout at a given time point in the VPCs may not add to 1. This is due to differences in time resolution between the ENDO and dropout assessments. The same is also true for PGA and dropout.

**Fig. 3:**
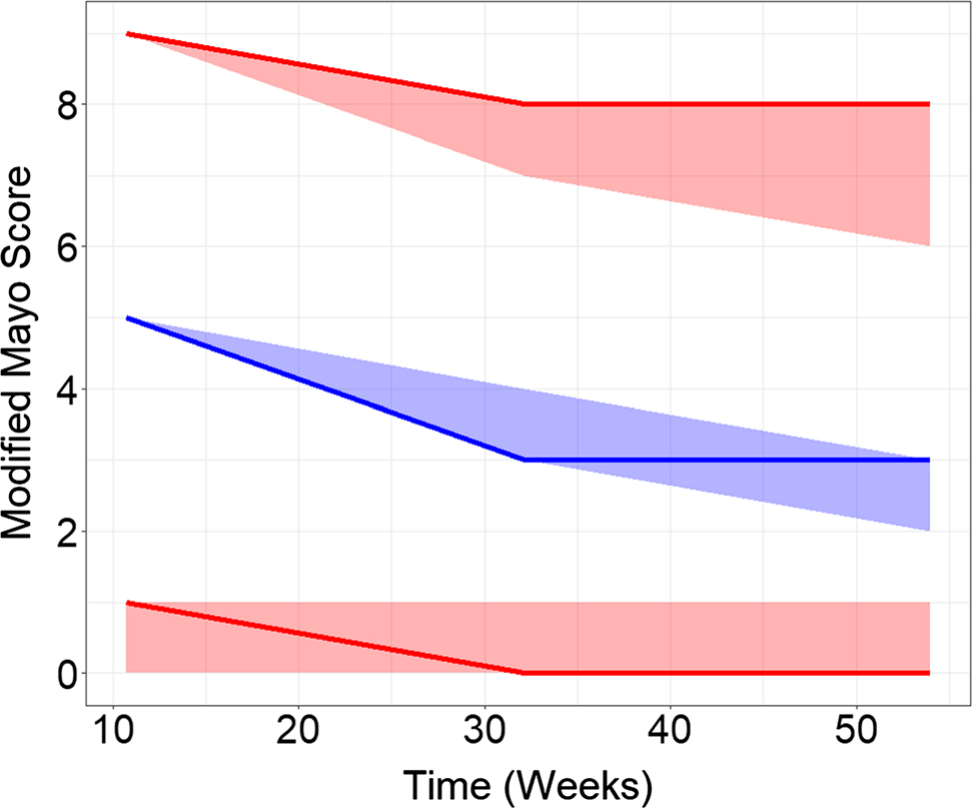
Continuous VPC of modified MCS over time in patients remaining in the trial. The median of the observed data is represented as a blue line, and the 2.5th and 97.5th percentiles are represented as red lines. Observed data are overlaid with shaded areas representing 95% prediction intervals by the model. Timepoints start at week 10 due to the first post-baseline ENDO assessment occurring during week 8–12 in the trials

The continuous VPC of the modified MCS showed agreement between the predicted 95% confidence interval and observed data. (Fig. 3) The modified MCS was derived by adding the RB + SF and ENDO estimates at each timepoint. Observed and predicted timepoints demonstrated a decrease in both scores over time. It should be noted that average scores at each timepoint were calculated using only scores from patients remaining in the trial. The dropout of patients (who generally had higher subscores) appears to be a large driver of the decrease in modified MCS over time. A comparison of model simulations with dropout and with no dropout of patients demonstrates that the decrease in the modified MCS over time is partly driven by the placebo response, and largely driven by the dropout of patients with higher modified MCS from the placebo/SoC arm (Fig. S1).

## Discussion

An ordered categorical model was developed to describe the modified MCS and MCS over time in placebo/SoC treated patients with moderate to severe active UC. While the primary objective was to model the modified MCS, modeling of the PGA subscore was explored to allow the model to estimate the MCS over time. RB and SF subscores were combined and modeled as a single endpoint, as the combination of these scores is a good representation of the symptomatic outcome at each time point. These two subscores are also typically assessed at the same timepoints in clinical trials. There is limited utility gained by separating the subscores in the current model; and combining the two subscores also allows for a more simple model structure. The ENDO subscore was estimated as a separate endpoint due to the limited number of ENDO assessments. The model estimates probabilities for the ENDO subscore categories at post-baseline timepoints based on the baseline ENDO subscore and RB and SF subscores from the same visit. The effect of the RB + SF covariate on the ENDO subscore model was statistically significant, and the model is able to produce predictions that agree well with the time course of the observed ENDO data (Fig. 2). The change from baseline in ENDO subscore, and therefore the placebo response in the subscore, can be explained by the time-course of RB and SF without the need for an additional parameter for time. In the current dataset, it appears that the change from baseline in the ENDO subscore, may hence be predicted using only the non-invasive RB and SF subscore evaluations.

The primary objective of this analysis was to model the modified MCS due to a number of considerations associated with the PGA subscore. The PGA subscore is subjective, it is unclear what information it provides that is distinct from the other subscores, and regulatory agencies have recently recommended against its use in clinical trials. While the PGA subscore can be evaluated at frequent timepoints in a clinical trial, the PGA subscore is assessed based on several factors including endoscopic evaluation. Therefore, the PGA subscore model in the current study only estimates this subscore at timepoints in which the ENDO subscore is measured. The need for an additional linear placebo effect to prevent overprediction of the PGA subscore model indicates the PGA subscore is affected to a greater extent by the placebo effect than the other subscores, and that the change from baseline in PGA subscore cannot be estimated from the modified MCS alone. This may be explained by the subjective and variable nature of the PGA subscore. Model misspecification of the PGA subscore may be affected by this subjectivity, and may also be due to the linkages between the subscore and dropout models. Any misspecifications in the RB + SF or ENDO subscore models, or dropout model can affect the PGA subscore model predictions. It should also be noted that the impact of RB + SF and the modified MCS on the subscore and dropout models was based on a continuous slope intercept model. This allowed for a more parsimonious approach involving less model parameters than if the scores were evaluated as categorical covariates on the subscore and dropout models.

In the covariate analysis, patients with prior treatment with TNF-α antagonists had a higher post-baseline RB + SF subscore over time than patients who were naïve to TNFα antagonists. It should be noted that patients with prior treatment with TNF-α antagonists had similar baseline values of RB and SF subscores as those that were naive to treatment with TNF-α antagonists. The covariate effect is consistent with the reported difficulty in treating patients with prior treatment with TNF-α antagonists [18]. This should be interpreted with caution, however, as the majority of data for patients with prior treatment with TNF-α antagonists came from a single study in the current dataset (Table 1). Notably, patient-related factors of age, baseline CRP, baseline serum albumin level, and smoking status, and study-level differences in the proportion of baseline steroid use and in the protocol specifications related to concomitant medications did not have a significant impact on the RB + SF subscore. The lack of age effect may be resulting from the lack of pediatric data, as the current dataset is limited to only adult patients. The effect of steroids and concomitant medications may be difficult to identify due to the lack of patient-level concomitant medication data.

The findings of the dropout model, where patients with higher RB + SF subscores had an increased probability of dropping out at the end of induction, are aligned with the common practices in clinical trials of re-assigning or re-randomizing only patients who responded to the assigned treatment at the beginning of the maintenance phase, and of providing rescue treatment to patients who do not respond. Responder status is typically defined by components of the MCS, where patients with higher scores are nonresponders, and those reaching defined lower scores are responders. The dropout model appears to slightly overpredict the dropout of patients at the end of induction, and this is likely due to the variety in mechanisms by which patients are removed from placebo/SoC arms in the clinical trials. For simulations of a single trial with set criteria for dropout, this could potentially be mitigated by mirroring these criteria in the model structure. In the current dataset, because there were no flags provided to differentiate between patients who drop out according to protocol criteria from those dropping out for other reasons, a general logistic regression structure (Eq. 7) was employed (similar to what has been reported previously to account for the dropping out from tumor measurement follow-up [19]) accounting for the correlation between lack of efficacy and probability of dropping out. The drop-out is assumed to be driven by the observed score and not in itself be informative of the response. Because dropout is correlated with efficacy outcomes, it will influence the observed efficacy time course and needs to be accounted for in the modeling process. This allows for simulation based evaluations of the model (VPCs) and an understanding of the contribution of drop-out to the observed change in MCS over time. A simple model structure without covariates was considered adequate for the current modeling process as the purpose of the drop-out model was to ensure that adequate VPCs could be generated accounting for the correlation between drop-out and efficacy outcomes in the simulations. It was also assumed that the primary driver of dropout was lack of efficacy. The dropout model was driven by the RB + SF subscore due to the frequent assessment of this subscore. Requiring the ENDO or PGA subscore, in the current model structure, would limit the observations informing dropout to very few timepoints.

To our knowledge, this is the first study to model the longitudinal MCS using linked models for MCS subscores and dropout. Hu et al. had previously developed an ordered categorical model, which estimated the MCS as a single endpoint [11]. Modeling the data sequentially with separate PO models provides the flexibility to estimate and understand the behavior of individual subscores over time. The RB + SF subscore and PGA subscore models, for example, may be used to predict the partial MCS and interpret early clinical trial data or for interim analyses, when ENDO subscores may not be available. With the complete model, various combinations of MCS subscores such as the modified or partial MCS, can be estimated. It should be noted that the linked model structure does not intend to suggest causality between subscores. Rather, the model structure was developed based on the assumption that each subscore reflects disease progression, and therefore, may inform other subscores. The development of the dropout model allowed for the model to capture the complexity in UC clinical trial design, where placebo/SoC arm patients may or may not remain in the placebo/SoC arm during the maintenance phase. As seen in Fig. S1, accounting for this aspect of trial design is needed for accurate estimates of the MCS during the maintenance phase. Modeling the dropout of patients, in addition to the subscores, can also inform sample size estimation in clinical trial design. Alternative model structures (e.g. item response theory, Markov models) were considered for this analysis, but the PO model was selected. While an item response theory model may allow for simultaneously fitting different subscores to a shared underlying disease progression, the current model structure still acknowledges the correlation between the subscores. The PO model has the most simple structure, and describes the current data well. More complex models were therefore not considered necessary for the current analysis. Alternative linkages between the subscore models were also explored. A linkage in which subscore models were informed by the model-predicted cumulative probability of RB + SF ≥ 1 rather than the observed RB + SF subscore or modified MCS was evaluated. This would allow for ENDO and PGA subscores to inform parameters for the RB + SF subscore model. However, this did not improve the model fit, and the current approach is favored due to the easier potential application to future trials by directly predicting components of the MCS based on observed RB + SF subscores alone.

Key limitations in the current model should be considered. The data only included patients who had moderate to severe active UC at baseline. The developed model should therefore not be used to predict outcomes in patients with mild UC. IIV could not be estimated on the linear slope parameter due to insufficient data. The effect of intrinsic and extrinsic factors on disease progression, therefore, could not be evaluated. Because there are no Markov elements to explicitly account for the relationship between serial observations, the model is not suited for individual-level predictions. The aim of the current model was to generate summary-level predictions of the modified MCS over time, however, and the incorporation of a Markov element was not considered to be necessary. The model is unable to predict baseline values for the subscores, because baseline values are included as covariates in the subscore models. The inclusion of the observed baseline score as a covariate in the model is useful when the distribution of baseline scores is non-normal or cannot easily be transformed to a normal distribution [20]. This was the case in the present data due to the inclusion criteria in the clinical trials related to baseline MCS (e.g. subject must have a MCS of 6–12 points and an endoscopy subscore of ≥ 2). The developed model allows for predictions of the longitudinal MCS for other trials based on an assumed distribution of baseline scores e.g. similar to the current data set as shown in Table 1 or based on the expected distribution of the baseline score in the population of interest. All of the clinical trials included in the current study were 10 years or older, and trial design and conduct may not be consistent with more recent clinical trials. In particular, the included trials are unlikely to have used central endoscopies, and therefore the ENDO data is likely more variable than the data from more recent trials. The standard of care has also changed for UC over time, as new therapeutic agents have become available. Placebo/SoC arm patients in more recent clinical trials are thus more likely to have been treated with TNF-α antagonists and other newer therapies than patients from older trials. Because patient-level data for concomitant medications could not feasibly be formatted for modeling, the effect of concomitant medications could only be evaluated using information from the study protocols on the minimum length of treatment prior to study and the medications permitted in the study. In addition, the RB + SF subscore may be calculated in a trial using a variety of methods (e.g. worst score in a time interval, average score in a time interval), which may contribute to variability in the RB + SF subscores in the current dataset [21]. Because the methods used across all of the studies could not be determined, the effect of various calculation methods on the RB + SF subscore model was not evaluated in the model.

The current model presents a case example for use of the historical trial data provided by TransCelerate Biopharma. TransCelerate’s data sharing initiative promotes the use of historical trial data to improve efficiency in drug development. Their historical control database is a collaborative platform with member companies actively contributing de-identified, patient-level clinical trial data [5]. As the database grows and incorporates data from more recent trials, the use of historical data can be improved and explored in other indications as well. Outside of disease modeling, the database can be used for additional cases including safety signal interpretation and biomarker development.

Future directions would involve incorporating additional, more recent clinical trial data to update the model. Considering the additional challenges in pediatric drug development for UC, the availability of pediatric data would also allow for a better understanding of age effects on placebo response. A robust model can generate predictions that can be used as a “virtual placebo arm” for pediatric trials in which a placebo/SoC arm is not feasible to enroll. The model could also have a drug effect parameter incorporated into it, so that it may be applied to data from treatment arm patients. Drug effect may be evaluated categorically, by including separate slopes for the linear effect (Effect =  *− *SLOPE × TIME) for patients in the treatment and placebo arm. If an examination of the concentration–response relationship was of interest, observed or population PK model predicted values may be accounted for in the current model using various approaches such as an effect compartment or indirect response model. The current study pooled data from five clinical trials to develop a longitudinal model describing the modified MCS over time in placebo/SoC arm patients with moderate to severe active UC. By providing insights into the time course of placebo response, and factors that influence the response, the model can support clinical trial design and data interpretation.

## Supplementary Information

Below is the link to the electronic supplementary material.

Supplementary file1 (DOCX 677 kb)
