# Supplementary material for: A longitudinal model for the Mayo Clinical Score and its sub-components in patients with ulcerative colitis
Source: J Pharmacokinet Pharmacodyn. 2021 Oct 16;49(2):179–90. doi: 10.1007/s10928-021-09789-2 (PMC8940756; doi:10.1007/s10928-021-09789-2)
Supplement: Supplementary file 1 — Supplementary file1 (DOCX 677 kb) [file 10928_2021_9789_MOESM1_ESM.docx]

**Supplementary Figures**

**Fig. S1** Comparison of model predicted modified MCS with dropout (red) and without dropout (blue). Shaded areas represent 95% confidence intervals.


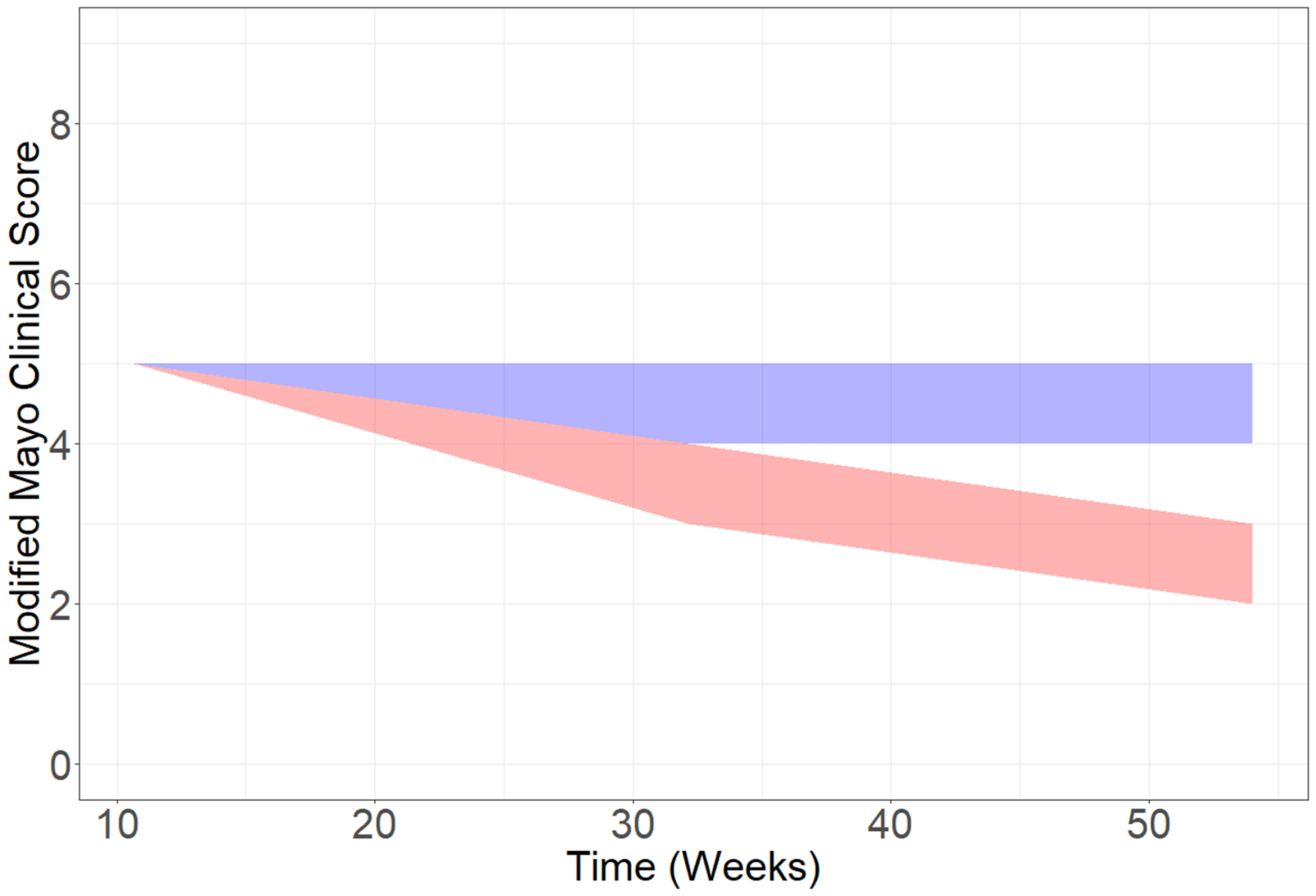


**Fig. S2** Continuous VPC of the MCS over time in patients remaining in the trial. The median of the observed data is represented as a blue line, and the 2.5th and 97.5th percentiles are represented as red lines. Observed data are overlaid with shaded areas representing 95% confidence intervals by the model. The MCS was derived by adding the RB+SF, ENDO, and PGA subscore model estimates at each timepoint. Timepoints start at week 10 due to the first post-baseline ENDO assessment occurring during week 8-12 in the trials.

**
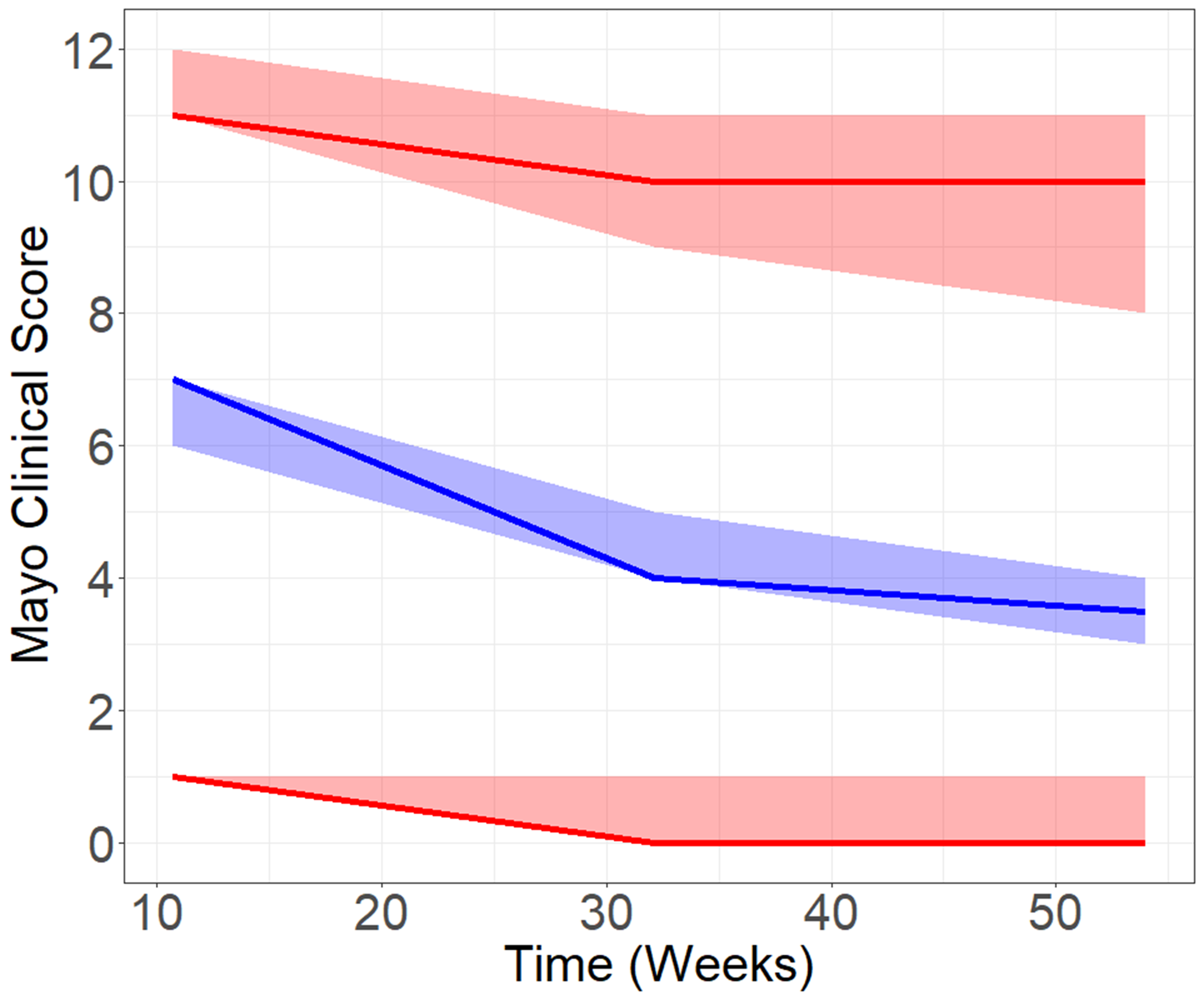
**
